# Biological Characterizations of H5Nx Avian Influenza Viruses Embodying Different Neuraminidases

**DOI:** 10.3389/fmicb.2017.01084

**Published:** 2017-06-14

**Authors:** Yuandi Yu, Zaoyue Zhang, Huanan Li, Xiuhui Wang, Bo Li, Xingxing Ren, Zhaoyong Zeng, Xu Zhang, Shukai Liu, Pingsheng Hu, Wenbao Qi, Ming Liao

**Affiliations:** ^1^National and Regional Joint Engineering Laboratory for Medicament of Zoonosis Prevention and Control, College of Veterinary Medicine, South China Agricultural UniversityGuangzhou, China; ^2^Key Laboratory of Zoonosis, Key Laboratory of Animal Vaccine Development, Ministry of AgricultureGuangzhou, China; ^3^Key Laboratory of Zoonosis Prevention and Control of Guangdong Province, Ministry of AgricultureGuangzhou, China

**Keywords:** H5Nx, highly pathogenic avian influenza virus, neuraminidase, evolution epidemic, pathogenicity

## Abstract

The H5 subtype virus of Highly Pathogenic Avian Influenza Virus has caused huge economic losses to the poultry industry and is a threat to human health. Until 2010, H5N1 subtype virus was the major genotype in China. Since 2011, reassortant H5N2, H5N6, and H5N8 viruses were identified in domestic poultry in China. The clade 2.3.4.4 H5N6 and H5N8 AIV has now spread to most of China. Clade 2.3.4.4 H5N6 virus has caused 17 human deaths. However, the prevalence, pathogenicity, and transmissibility of the distinct NA reassortment with H5 subtypes viruses (H5Nx) is unknown. We constructed five clade 2.3.4.4 reassortant H5Nx viruses that shared the same HA and six internal gene segments. The NA gene segment was replaced with N1, N2, N6, ΔN6 (with an 11 amino acid deletion at the 58th to 68th of NA stalk region), and N8 strains, respectively. The reassortant viruses with distinct NAs of clade 2.3.4.4 H5 subtype had different degrees of fitness. All reassortant H5Nx viruses formed plaques on MDCK cell monolayers, but the ΔH5N6 grew more efficiently in mammalian and avian cells. The reassortant H5Nx viruses were more virulent in mice as compared to the H5N2 virus. The H5N6 and H5N8 reassortant viruses exhibited enhanced pathogenicity and transmissibility in chickens as compared to the H5N1 reassortant virus. We suggest that comprehensive surveillance work should be undertaken to monitor the H5Nx viruses.

## Introduction

Influenza A virus (IAV) belongs to the *Orthomyxoviridae* family of RNA viruses that have negative-sense, single-stranded, and segmented RNA genomes. IAVs are classified into subtypes based on antibody responses to the two major viral surface glycoproteins, hemagglutinin (HA) and neuraminidase (NA) (Yoon et al., 2014). Sixteen HA subtypes (H1–H16) and nine NA subtypes (N1–N9) are circulating in birds (Fouchier et al., 2005). The A/goose/Guangdong/1/1996 (H5N1) subtype virus was first isolated in Guangdong Province of China in 1996 and has posed threat to public health (Duan et al., 2007). To date, 856 people have been infected with the H5N1 subtype virus and 452 people have died since 2003^1^. IAV continues to evolve and clade 2.3.4.4 IAVs with other NA subtypes (N2, N6, and N8) are now prevalent (Lee et al., 2015; Pasick et al., 2015; Su et al., 2015; Claes et al., 2016). H5N2 and H5N8 viruses spread from migratory birds to poultry in multiple continents in 2014 (Verhagen et al., 2015; Lycett et al., 2016). Humans can be infected with the H5N6 subtype avian influenza virus (Chen and Zhang, 2015; Yang et al., 2015). These H5Nx viruses (H5N1, H5N2, H5N6, and H5N8) seriously threaten human health and the poultry industry.

Influenza A virus attaches to cellular membranes via HA interacting with sialic acids expressed at the terminal position of carbohydrate chains of cell surface glycoproteins and glycolipids (Matrosovich et al., 1997). NA activity catalyzes the removal of sialic acids from viral and cellular components, promotes the release of viral progeny, and prevents virion aggregation (Ruangrung et al., 2016). NA reassortment with H5 subtype viruses in the evolution of H5N1 avian influenza virus leads to novel combinations of H5Nx (H5N2, H5N6, and H5N8) subtypes (Claes et al., 2016). The balance between HA and NA proteins contributes to expression of the HPAI phenotype (Diederich et al., 2015). Furthermore, the NA gene has been shown to play an important role in influenza virus enzyme activity, transmission, and pathogenicity (Lv et al., 2015; Stech et al., 2015; Ranadheera et al., 2016).

Structural analyses of the H5Nx NAs (N1, N2, N6, and N8) indicated that the overall NA structures are very similar (Yang et al., 2016). Using next-generation sequencing (NGS), virus isolation methods and genetic evolution, H5N6 has been confirmed to have replaced H5N1 as one of the dominant subtypes among poultry in southern China (Bi et al., 2016; Li et al., 2017). In addition, an analysis of how 2.3.4.4 H5Nx viruses had spread in the geographical and environmental space was carried out using Boosted Regression Tree (BRT) models (Dhingra et al., 2016). However, the biological characteristics of distinct NAs and functional comparisons with the 2.3.4.4 H5 HA subtype viruses are not well clarified. To study the pandemic potential, NA biological characteristics, and genetic interactions between HA and NA of the clade 2.3.4.4 H5Nx virus, we constructed and rescued five reassortant H5Nx viruses. A clade 2.3.4.4 H5N1 virus, A/goose/Guangdong/SH7/2013 was used as the genetic backbone and the N1 gene segment was replaced with N2, N6, ΔN6 (with an 11 amino acid deletion at the 58th to 68th in NA stalk region), and N8 NAs using reverse genetics. We then characterized the biology of these viruses in mammalian and avian cells, determined their virulence in mice and chickens, and characterized their horizontal transmission in chickens.

## Materials and Methods

### H5Nx Influenza Virus Analyses

All sequences of H5Nx viruses (H5N1, H5N2, H5N6, and H5N8) from 1996 to 2016 in China that were available from the GISAID and GenBank databases were downloaded. And the sequences of H5N6 virus aligned using the MegAlign 6.06 program (DNASTAR) with the ClustalW method.

### Viruses and Cells

The clade 2.3.4.4 H5 subtype avian influenza viruses, including A/goose/Guangdong/SH7/2013 (H5N1) (SH7, EPI_ISL_259927), A/duck/Guangdong/673/2014 (H5N6) (673, EPI_ISL_259923), A/duck/Guangdong/674/2014 (H5N6) (674, EPI_ISL_259924), and A/goose/Jiangsu/1306/2014 (H5N8) (Js1306, EPI_ISL_259926) were isolated from sick ducks or geese. The H5N2 avian influenza virus, A/chicken/Hebei/LZF/2014 (H5N2) (LZF, EPI_ISL_259925) was isolated from a sick chicken. All viruses were propagated in 10-day-old SPF embryonated eggs at 37°C and frozen at -80°C. Human embryonic kidney cells (HEK293T), Madin-Darby canine kidney (MDCK), and Chicken Embryo Fibroblast (CEF) cells were grown in Dulbecco’s modified essential medium (DMEM; Gibco) with 10% fetal bovine serum (FBS; BI) and 1% penicillin/streptomycin (Gibco).

### Plasmids Construction and Generation of Reassortant Viruses

N1 gene segments were replaced with NAs from LZF, 673, 674, and Js1306, respectively, and were cloned into a pHW2000 plasmid system according to a previous study (Hoffmann et al., 2000; Xiao et al., 2016). HEK293T cells monolayers in 6-well plates were transfected at 80–90% confluency with 4 μg of the eight plasmids (500 ng of each plasmid) by using Lipofectamine 3000 (Invitrogen) according to the manufacturer’s instructions. Six hours later, the mixture was replaced with Opti-MEM (Gibco) containing 0.2% bovine serum albumin and 1 μg/ml TPCK trypsin (Sigma). After 48 h, the supernatant was harvested and injected into SPF embryonated eggs for virus propagation. Viruses were titrated in embryonated eggs using hemagglutination assays and sequenced. The five reassortant H5Nx viruses were named rH5N1, rH5N2, rH5N6, ΔrH5N6, and rH5N8.

### Virus Plaque Assay

The plaque assay was adapted from previously described procedure (Tian et al., 2012). MDCK cells were grown in DMEM with 1 mM L-glutamine and 10% FBS and seeded onto 6-well plates. Confluent monolayers were washed twice with phosphate-buffered saline (PBS) and infected with serial 10-fold dilutions of the virus at 37°C. After 2 h of incubation, the cells were washed twice with PBS and then overlaid with MEM containing 1% agarose. After 48–72 h of incubation at 37°C, the agarose was removed and cells were stained with 0.5% crystal violet in 10% formaldehyde solution. The plaques were visualized and manually counted.

### Hemagglutination and Hemagglutination-Elution Assays

The balance between HA binding and NA cleavage was measured by performing hemagglutination assays as previously described (Jean-Sébastien Casalegno, 2014). Viruses were prepared using serial twofold dilutions in 50 μl of PBS in 96-well plates and incubated with 50 μl of 0.8% guinea pig erythrocytes at 4°C for 75 min and then at 37°C for 2 h. HA titers were recorded at both temperatures, respectively. Each sample was assayed in triplicate.

### Neuraminidase Activity Assay

A fluorescent substrate 2′ (4-methylumbelliferyl)-α-D-*N*-acetylneuraminic acid (MUNANA; Sigma) was used to measure NA enzymatic activity, according to the manufacturer’s recommendations. Viruses were prepared using serial twofold dilutions in 50 μl of calcium saline buffer in 96-well plates and 50 μl of 200 μmol MUN was added to each well, and then incubated for 60 min at 37°C in darkness. One hour later, stop solution was added and NA activity was quantified using a Synergy HT Multi-Detection microplate reader (BioTek, Winooski, VT, United States) with excitation and emission wavelength of 360 and 440 nm.

### Virus Growth Kinetics

Confluent MDCK cells were infected at a multiplicity of infection (MOI) of 0.001 TCID_50_/cell, CEF cells were infected at MOI of 0.0001 TCID_50_/cell for 1 h at 37°C on 6-well plates, as described previously (Xiao et al., 2016). One hour later, plates were washed twice with PBS, and then incubated with DMEM containing 0.2% BSA at 37°C or 39°C with 5% CO_2_. Culture supernatants were collected at 12, 24, 36, and 48 hour post-inoculation (h.p.i.). The virus titers were determined by performing 50% tissue culture infective dose (TCID_50_) assays in MDCK cells.

### Animal Experiments

Groups of eight female 4- to 6-week-old BALB/c mice (Vitalriver Company, Beijing, China) were anesthetized with CO_2_ and inoculated intranasally (i.n.) with 10^5^ EID_50_/50 μl of the five reassortant H5Nx viruses (rH5N1, rH5N2, rH5N6, ΔrH5N6, and rH5N8). Body weight and clinical symptoms were monitored daily for 14 days after infection. The mice were euthanized if they lost more than 25% of their initial body weight. Three mice from each group were euthanized on 4 days post-inoculation (d.p.i.), and the lung, brain and turbinate tissues were collected to determine the virus titers by EID_50_ assay. The 50% mouse lethal dose (MLD_50_) were determined by inoculating groups of five mice with serial 10-fold dilutions of virus ranging from 10^1^ to 10^5^ EID_50_/50 μl. The MLD_50_ values were calculated using the Reed and Muench method.

Groups of seven 5-week-old SPF chickens (Merial Vital Company, Beijing, China) were inoculated intranasally with 10^5^ EID_50_/200 μl of five reassortant H5Nx viruses, respectively. Seven uninfected chickens served as negative controls. All chickens were observed for clinical symptoms for 14 days. On 1 d.p.i., seven naive chickens (contact) were mixed with treatment group chickens. Cloacal and throat swabs were collected on 3, 5, and 7 d.p.i. from infected and naive chickens. Three inoculated chickens in each group were euthanized on 3 d.p.i. to test the virus replication in organs, including heart, lung, kidney, brain, spleen, and liver. Similar actions were performed on chickens if they died within 3 days. For the chickens in the naive contact group, the virus replication in heart, lung, kidney, brain, spleen, and liver were detected after the chickens died.

### Ethics Statement and Biosafety

All experiments were carried out in ABSL-3 facilities in compliance with the biosafety committee of South China Agriculture University (SCAU) protocols. All animal experiments were reviewed and approved by the Institutional Animal Care and Use Committee at SCAU and were carried out in accordance with the approved guidelines.

## Results

### Evolution and Epidemiology of H5Nx Influenza Viruses in China

**FIGURE 1:**
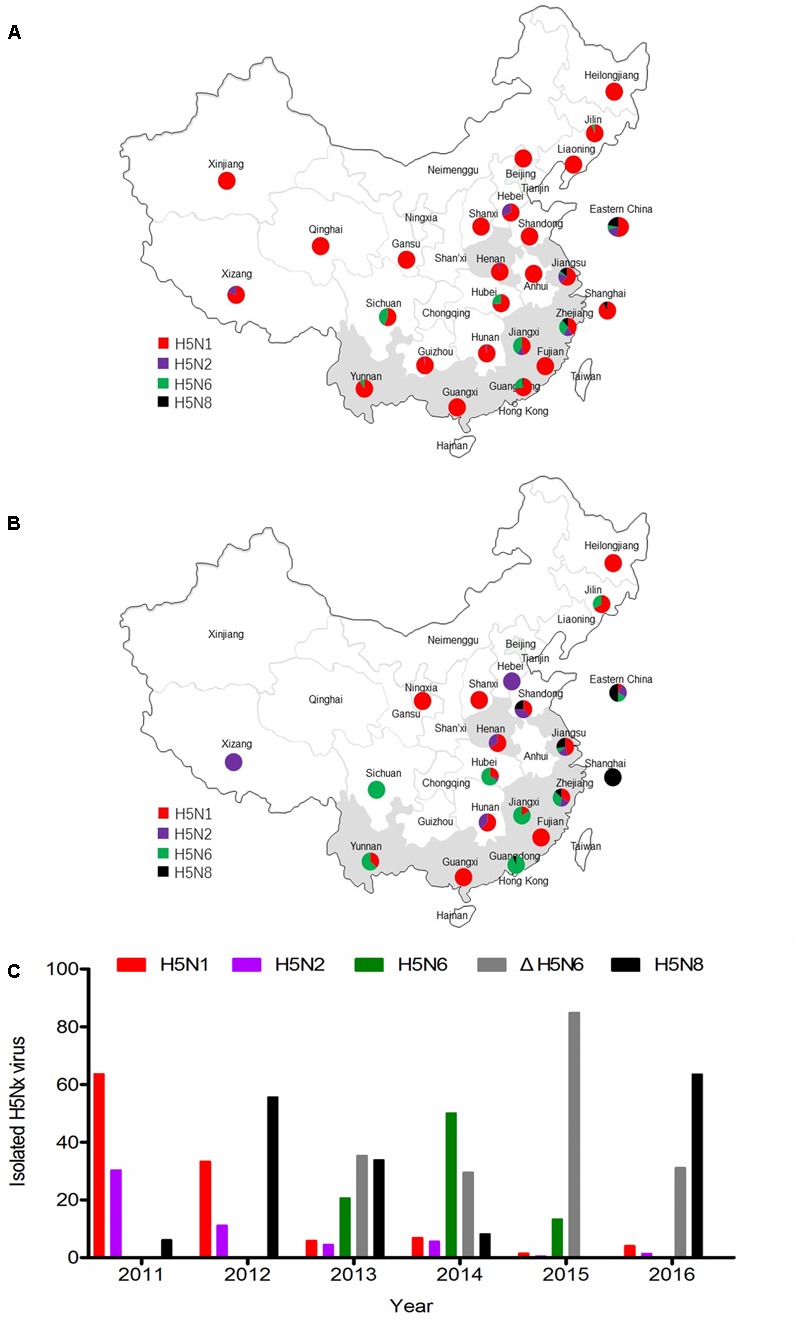
Epidemiology and distribution of H5Nx avian influenza viruses in China. **(A)** Distribution of H5Nx AIVs in China 1996–2010. **(B)** Distribution of H5Nx AIVs in China 2011–2016. **(C)** The isolation rate of H5Nx viruses isolated in China. Data are from NCBI (https://www.ncbi.nlm.nih.gov/genome/viruses/variation/flu/) and GISAID (http://platform.gisaid.org/epi3/frontend). Colors depict different virus subtypes. H5N1, H5N2, H5N6, ΔrH5N6, and H5N8 are shown in red, purple, green, gray, and black, respectively.

Until 2010, H5N1 subtype virus was the major genotype in China (**Figure 1A**). The H5N2 and H5N8 viruses were sporadically reported in China after 2011. The H5N6 virus was isolated in 2013 and became a major genotype in China. The clade 2.3.4.4 viruses were detected in most regions of China from 2013 to 2016 (**Figures 1B,C**). In addition, H5N2 virus was mainly isolated in northern China. The NA6 stalk region can be divided into two groups according to the presence of an amino acid deletion in the positions 58–68 of NA stalk region. The percentage of 11 amino acids deletion in NA stalk region of H5N6 viruses were 63% (24 of 38 viruses), 37% (69 of 186 viruses), 87% (359 of 408 viruses), and 100% (23 of 23 viruses) respectively, from 2013 to 2016 (**Figure 1C**). Seventeen H5N6 strains of clade 2.3.4.4 have caused human infections^2^. Sixteen viruses had the 11 amino acid deletion at the 58th to 68th in the NA stalk region. The H5N8 virus has become the pandemic genotype in 2016 (**Figure 1C**).

### HA and NA Properties of H5Nx Reassortant Viruses

**FIGURE 2:**
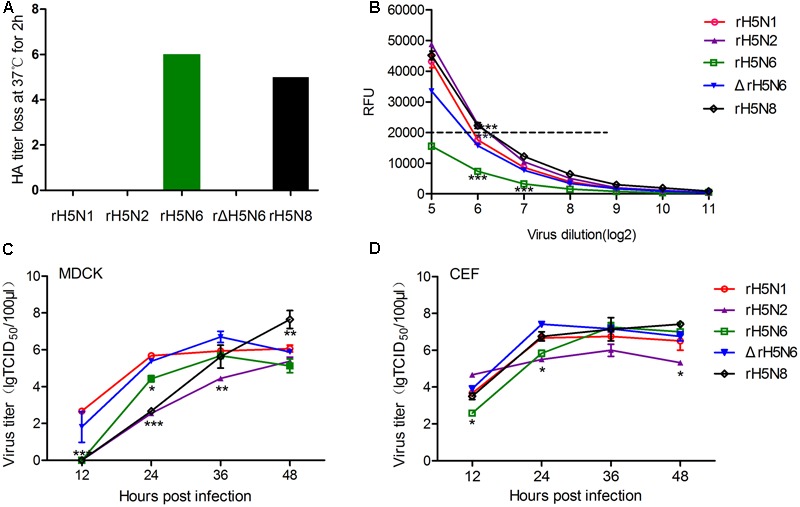
Hemagglutinin (HA)-neuraminidase (NA) properties and growth kinetics of H5Nx reassortant viruses. **(A)** Elution assays of guinea pig RBCs. H5Nxreassortant viruses were serially diluted in phosphate-buffered saline (PBS) and incubated with 0.8% RBC suspensions at 4°C for 75 min, then 37°C for 2 h. And then the loss of HA activity was recorded. Each virus was analyzed in triplicate wells. **(B)** NA activity was measured by a solution assay using the fluorescence substrate 4-MU-NANA. NA activity was represented by the fluorescence intensity represent the NA activity (^∗∗^*P* < 0.01, ^∗∗∗^*P* < 0.001). **(C)** Madin-Darby canine kidney (MDCK) cells were inoculated with viruses at an multiplicity of infection (MOI) of 0.001. **(D)** Chicken Embryo Fibroblast (CEF) was inoculated with viruses at an MOI of 0.0001. Samples were collected at 12, 24,36, and 48 hour post-inoculation (h.p.i.). Virus titers were determined by using the 50% tissue culture infectious dose (TCID_50_) assay. The virus titers are means ± standard deviations (SD) (*n* = 3), Statistical significance was analyzed using an unpaired *t*-test: ^∗^*P* < 0.05, ^∗∗^*P* < 0.01.

To directly measure the balance between HA and NA, the loss of HA titer at 37°C was compared to the erythrocyte agglutination rate at 4°C. The binding and release of five reassortant viruses were quantitatively measured via guinea pig erythrocytes. At 4°C, the NA is inactive while HA can bind to its receptors and agglutinate the erythrocytes. At 37°C, the NA becomes active; thus, the loss of HA titer evaluates the equilibrium between HA affinity and NA activity. HA titers were reduced by five- and sixfold for the rH5N6 and rH5N8 viruses, respectively. The rH5N1, rH5N2, and ΔrH5N6 viruses did not show any change of HA titer at either temperature (**Figure 2A**). 2′ (4-methylumbelliferyl)-α-D-*N*-acetylneuraminic acid was used to evaluate the NA activities of five H5Nx reassortant virus (Potier et al., 1979). The rH5N2 and rH5N8 viruses had RFU values, and the rH5N6 and ΔrH5N6 viruses had lower activities, as compared to rH5N1 (**Figure 2B**).

### Plaque-Forming Ability and Replication Kinetics of Reassortant H5Nx Viruses

**FIGURE 3:**
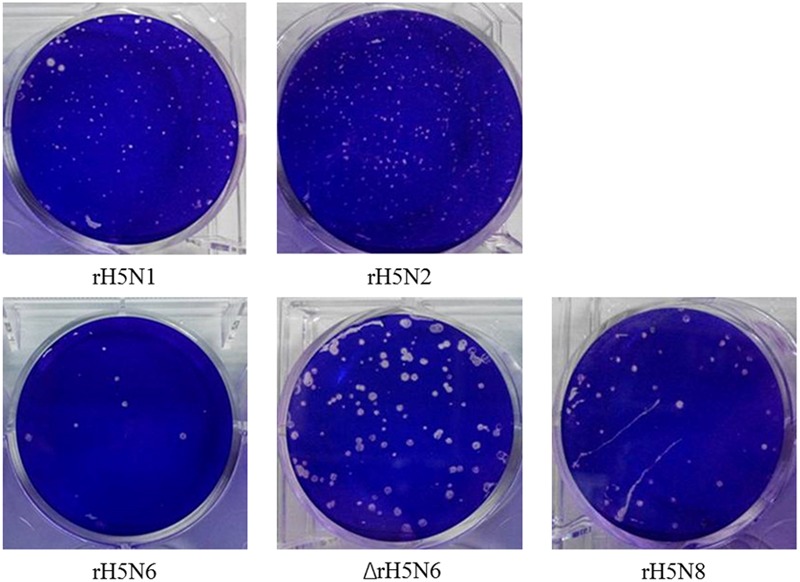
Plaque-forming ability of H5Nx reassortant viruses in MDCK cell monolayers. Cells were infected with rH5N1, rH5N2, rH5N6, ΔrH5N6, and rH5N8 virus. At 2 h.p.i., cells were washed twice with PBS and then overlaid with MEM containing 1% agarose. After 48–72 h of incubation at 37°C, plaques were counted.

We then characterized the cleavage properties of the HA protein combined with different NAs by performing plaque assays. All reassortant H5Nx viruses formed plaques on MDCK cells without TPCK trypsin. MDCK cells infected with rH5N8 virus grew the largest plaques (1.80 mm) when compared to rH5N6 and ΔrH5N6 viruses. The rH5N1 virus and rH5N2 virus formed pin-point plaques in MDCK cells (from 0.48 to 0.73 mm) (**Figure 3**).

The growth kinetics of reassortant H5Nx viruses on mammalian and avian cells (MDCK and CEF) were determined. In MDCK cells, the rH5N1 and ΔrH5N6 viruses replicated more efficiently and had the highest virus titers at 12, 24, and 36 h. By contrast, rH5N2 had the lowest virus titer at each time point. The rH5N6 and rH5N8 reassortant viruses exhibited an intermediate level of virus titers among H5Nx reassortant viruses. Growth kinetic of rH5N8 was performed using a significantly higher titer when compared to other reassortant H5Nx viruses at 48 h. And the H5N2 virus also appeared to surpass the rH5N6 virus at 48 h (**Figure 2C**). In avian CEF cells, ΔrH5N6 replicated more efficiently than the other reassortant H5Nx viruses. rH5N2 had the lowest virus titers at each investigated time point, except at 12 h. rH5N8 growth kinetics were similar to rH5N1 but higher than rH5N6 (**Figure 2D**). These results showed that the ΔrH5N6 grew efficiently in MDCK and CEF cells. In contract, rH5N2 virus had lower titer in these cells.

### Pathogenicity of H5Nx Reassortant Viruses in Mice

**FIGURE 4:**
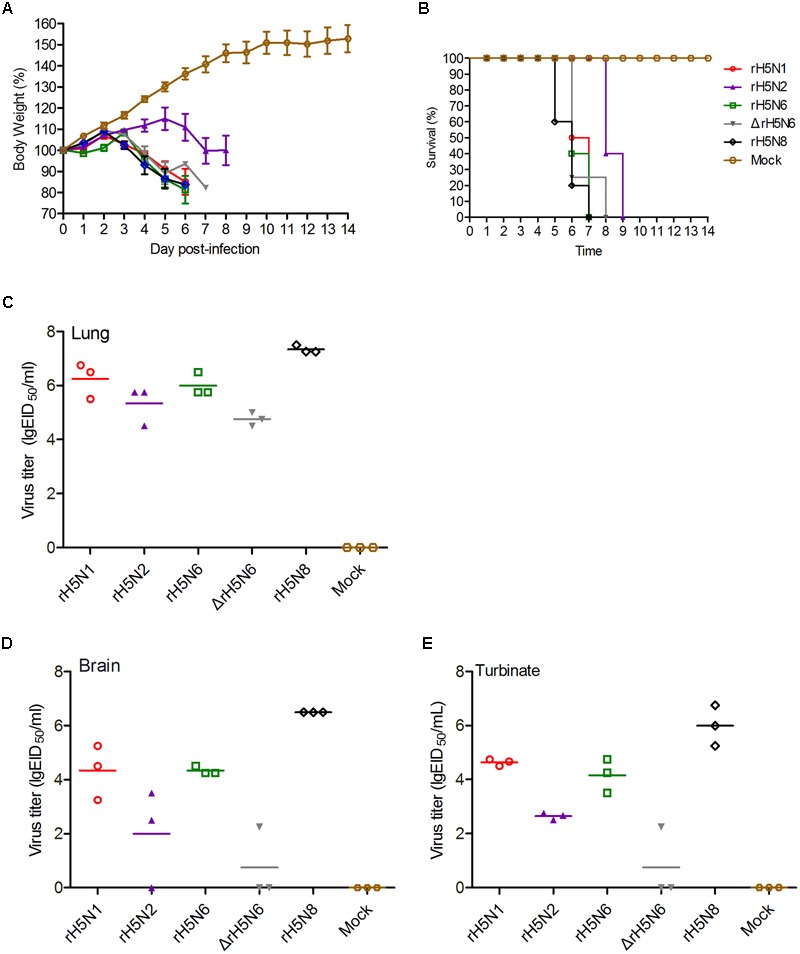
Pathogenicity of the of H5Nx reassortant viruses in mice. Groups of eight female BALB/c mice were intranasally inoculated with 10^5^ EID_50_/50 μl of the indicated viruses or with PBS as a negative control. Mouse body weight **(A)** and survival **(B)** were monitored daily for 14 days. Mice that lost more than 25% of their initial weight were euthanized. The percentage weight from each group and each time point are presented as means ± SD. The titers of viruses were detected in organs lung **(C)**, brain **(D)**, turbinate **(E)**. Three mice from each group were euthanized on 4 days post-inoculation (d.p.i.).

To investigate whether these five H5Nx reassortant virus also replicate efficiently in mammalian hosts, BALB/c mice were infected with 10^5^ EID_50_/50 μl of these H5Nx reassortant viruses. All viruses were lethal to mice with 2 d.p.i., except for rH5N2 (at 5 d.p.i.). Mice infected with rH5N1, rH5N6, ΔrH5N6, and rH5N8 died within 6 d.p.i., whereas mice infected with rH5N2 showed delayed fatality on 8 d.p.i. (**Figures 4A,B**). All viruses replicated efficiently in lung and nasal tissues. As H5N1 IAV can cause systemic infection, neurotropism, and long-term effects on the central nervous system (CNS) (Shinya et al., 2011), the replication of H5Nx reassortant viruses in mice brain was tested. The rH5N1, rH5N6, and rH5N8 virus were found in all tested mice brains, while rH5N2 and ΔrH5N6 were only detected in 2/3 or 1/3 mice, respectively (**Figures 4C–E**). Subsequently, to study the virulence of H5Nx reassortant viruses, BALB/c mice were inoculated with serial 10-fold dilutions of the virus to determine viral MLD_50_. The MLD_50_ (lgEID_50_) of rH5N1, rH5N6, ΔrH5N6, and rH5N8 were 2.5, 3.3, 2.68, 3.16, and 2.5, respectively.

### Pathogenicity and Transmission of H5Nx Reassortant Viruses in Chickens

**FIGURE 5:**
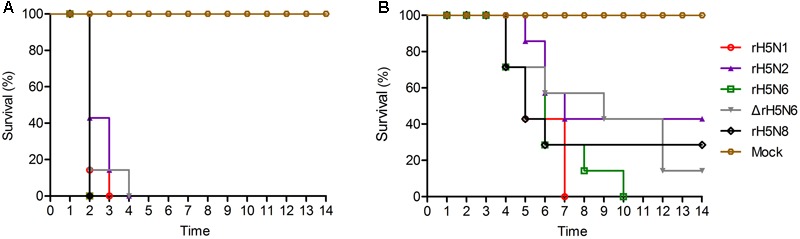
Pathogenicity and transmission of H5Nx reassortant viruses in chickens. Groups of seven 5-week-old SPF chickens were inoculated intranasally with 10^5^ EID_50_/200 μl of the indicated viruses. Seven chickens were used as negative controls. **(A)** Survival rate of chickens infected with H5Nx reassortant viruses. **(B)** Survival rate of chickens co-housed with the animals infected indicated virus. All chickens were observed for clinical symptoms for 14 days.

Groups of seven 5-week-old SPF chickens were inoculated intranasally with 10^5^ EID_50_/50 μl of the five H5Nx reassortants. Chickens infected with rH5N2 and ΔrH5N6 died within 4 d.p.i. ΔrH5N6, rH5N6, and rH5N8 viruses caused sudden death without symptoms of illness on 2 d.p.i. (**Figure 5A**). Reassortant viruses were isolated from lung, spleen, kidney, and brain samples at high virus titers. All H5Nx reassortant viruses replicated in lungs, with mean titers from 5.8 to 6.97 lgEID_50_. The ΔrH5N6 virus replicated highly in brains, with a mean titer of 6.58 lgEID_50_ (**Table 1**).

**Table 1:** Viral distribution of the H5Nx reassortant viruses in chicken tissues (lgEID_50_/200 μl).

Strains		Virus titers (lgEID_50_/200 μl)^a^ in					
		Heart	Liver	Spleen	Lung	Kidney	Brain
rH5N1	Infected^b^	3/3 (5.67 ± 1.16)	3/3 (5.75 ± 0.71)	3/3 (7.1 ± 0.28)	3/3 (5.9 ± 0.42)	3/3 (7 ± 0.89)	3/3 (5.75 ± 0.4)
	Contact^c^	3/3 (5.85 ± 0.28)	3/3 (6.0 ± 0.41)	3/3 (6.67 ± 1.17)	3/3 (5.8 ± 0.51)	3/3 (6.7 ± 0.69)	3/3 (5.58 ± 0.62)
rH5N2	Infected	3/3 (6.47 ± 0.22)	3/3 (6.25 ± 0)	3/3 (5.91 ± 1.2)	3/3 (6.97 ± 0.7)	3/3 (7.04 ± 0.43)	3/3 (5.94 ± 0.3)
	Contact	3/3 (7.2 ± 0.85)	3/3 (6.5 ± 0)	3/3 (6.07 ± 1.1)	3/3 (6.79 ± 0.3)	3/3 (6.77 ± 0.65)	3/3 (5.25 ± 0.32)
rH5N6	Infected	3/3 (5.45 ± 1.0)	3/3 (5.1 ± 0.39)	3/3 (5.92 ± 0.38)	3/3 (5.25 ± 0.5)	3/3 (6.64 ± 0.47)	3/3 (5.4 ± 0.47)
	Contact	3/3 (6.17 ± 0.28)	3/3 (6.25 ± 0.33)	3/3 (5.25 ± 0.68)	3/3 (6.36 ± 1.2)	3/3 (5.61 ± 0.51)	3/3 (5.5 ± 0.48)
ΔrH5N6	Infected	2/2 (5.75,7.55)	3/3 (6.5 ± 0.57)	3/3 (6.57 ± 0.29)	3/3 (6.69 ± 0.8)	3/3 (7.58 ± 0.39)	3/3 (6.58 ± 0.2)
	Contact	3/3 (6.49 ± 0.65)	3/3 (6.5 ± 0.57)	3/3 (6.8 ± 0.87)	3/3 (.8 ± 0.94)	3/3 (7.12 ± 0.75)	3/3 (5.67 ± 0.1)
rH5N8	Infected	3/3 (5.64 ± 0.69)	3/3 (5.25 ± 0.5)	3/3 (5.97 ± 0.48)	3/3 (5.8 ± 0.96)	3/3 (6.83 ± 0.76)	3/3 (5.9 ± 0.2)
	Contact	3/3 (5.9 ± 0.41)	3/3 (5.83 ± 1.2)	3/3 (6.71 ± 0.87)	3/3 (6.2 ± 0.93)	3/3 (7.19 ± 0.29)	3/3 (5.67 ± 0.28)
Mock		0/3	0/3	0/3	0/3	0/3	0/3

*^a^Seven 5-week-old SPF chickens were inoculated with 10^*5*^ EID_*50*_ of rH5N2, rH5N6, ΔrH5N6, and rH5N8 virus in a volume of 200 μl. Virus titers in hearts, livers, spleens, lungs, kidneys, and brains of the first three dead chickens, or of the euthanized infected chickens on 3 d.p.i. or of the naive contact chickens on 4 d.p.i. ^b^Chickens inoculated with virus. ^c^Naive contact chickens housed with inoculated chickens.*

To evaluate the transmission of five H5Nx reassortants in chickens, after 1 d.p.i., seven SPF chickens were co-housed with infected chickens. The naive contact chickens co-housed with chickens infected with rH5N1, rH5N6, ΔrH5N6, or rH5N8 viruses infection died at 4 d.p.i. Naïve chickens co-housed with chickens infected with the rH5N2 virus died on 5 d.p.i. (**Figure 5B**). No seroconversion was found in surviving contact animals. Oropharyngeal and cloacal swabs from contact chickens were tested at 3 d.p.i. or 5 d.p.i. No virus was detected in oropharyngeal among contact chickens at 7 d.p.i. (**Table 2**). The virus was detected in the lung, spleen, kidney, and brain of contact chickens (**Table 1**). Overall, the H5Nx reassortant viruses were highly pathogenic to chickens, and rH5N6, ΔrH5N6, and rH5N8 exhibited enhanced pathogenicity and greater transmissibility in chickens.

**Table 2:** Virus titers in chicken cloacal and oropharyngeal swabs^a^.

Strains		Cloacal swabs			Oropharyngeal swabs		
		3 d.p.i.	5 d.p.i.	7 d.p.i.	3 d.p.i.	5 d.p.i.	7 d.p.i.
rH5N1	Infected	–^b^	–	–	–	–	–
	Contact	0/7	2/4 (1.5, 2.3)	–	0/7	1/4 (3.5)	–
rH5N2	Infected	1/1 (3.2)	–	–	1/1 (3.6)	–	–
	Contact	0/7	1/6 (1.25)	0/3	1/7 (0.98)	1/6 (3.5)	0/3
rH5N6	Infected	–	–	–	–	–	–
	Contact	2/7 (1.5, 1.5)	1/5 (2.3)	0/2 (0)	2/7 (0.98, 1.5)	0/5 (0)	0/2 (0)
ΔrH5N6	Infected	1/1 (3.35)	–	–	1/1 (3.55)	–	–
	Contact	2/7 (1.5, 1.75)	1/5 (2.25)	0/4 (0)	2/7 (1.5, 1.75)	1/5 (2.5)	0/4 (0)
rH5N8	Infected	–	–	–	–	–	–
	Contact	2/7 (1.5, 0.97)	2/3 (2.5, 2.5)	0/2 (0)	2/7 (2.5, 1.75)	2/3 (2.25, 2.5)	0/2 (0)
Mock		0/7	0/7	0/7	0/7	0/7	0/7

*^a^Seven 5-week-old SPF chickens were inoculated with 10^*5*^ EID_*50*_ of rH5N2, rH5N6, ΔrH5N6, and rH5N8 virus in a volume of 200 μl. Cloacal and throat swabs were collected on 3, 5, and 7 d.p.i. from infected and naive chickens. ^b^The chickens have died out at sample collecting time point.*

## Discussion

The novel clade 2.3.4.4 H5Nx (H51N1, H5N2, H5N6, and H5N8) viruses present in migrating wild aquatic birds contributes to the circulation of avian influenza viruses. More recently, structural and biochemical analysis of clade 2.3.4.4 H5Nx virus as well as the emergence, spread, and persistence of H5Nx viruses have been well annotated (Claes et al., 2016; Sun et al., 2016; Yang et al., 2016). Our study focused on the pandemic H5Nx viruses of clade 2.3.4.4 embodying different NA genes and described the basic characteristics of viral virulence and transmission.

The NA activity balance HA activity was beneficial to viral entry and exit (Gen et al., 2013). NA with a stalk region that varies in length influences its enzymatic function and showed the different pathogenicity in chicken and mice (Zhou et al., 2009). In our study, ΔrH5N6 reassortant viruses was with the 11 amino acid deletion at the 58th to 68th in NA stalk region. In addition, previous studies indicated that the different NA genes with not the same pathogenicity of H9 influenza virus (Yan et al., 2016). The functional match between HA and NA is crucial to virus adaptation and evolution (Xu et al., 2012; Gaymard et al., 2016). rH5N2 and rH5N8 had higher NA activity as compared to the other H5Nx reassortants, while rH5N6 was inactive. Our results suggest that rH5N6 and rH5N8 may have a stable fitness between their HA and NA activities, which might explain why they have become prevalent viruses in China.

Hemagglutinin cleavage is a major determinant of avian influenza virus virulence (Horimoto and Kawaoka, 2001). Eurasian A(H5N8), North American A (H5N8), and North American A (H5N2) viruses were as susceptible to HA cleavage as HPAI H5N1 (Kaplan et al., 2016). The ability of rH5N2, rH5N6, ΔrH5N6, and rH5N8 viruses to form plaques were enhanced, as compared to rH5N1. Together, these data suggested that clade 2.3.4.4 HA of H5 subtypes combined with N2, N6, or N8 was beneficial to viral fitness. Furthermore, HA of H5 subtype combined with N6 and N8 virus grew more efficiently than N2, which demonstrated that the match of HA/NA enhanced virus replication, consistent with previous reports that HA and NA balance has a great impact on viral growth (Yen et al., 2011; Diederich et al., 2015).

H5N2 and H5N6 viruses are known to have increased virulence and replication efficiency in mice (Li et al., 2014, 2015). In our study, rH5N1, rH5N6, ΔrH5N6, and rH5N8 were highly virulent, while rH5N2 showed moderate virulence in mice (Katz et al., 2000). Virus titers were detected in the lungs of mice infected with the H5Nx reassortant viruses and organs from mice infected rH5N8 showed high titers. The ΔrH5N6 virus did not replicate in mice brain and nasal cavities. The H5N6 virus which has caused human infections has an 11 amino acids deletion at the 58th to 68th in the NA stalk region. However, the influence of this deletion in NA stalk region on virus transmission needs to be investigated further.

Our findings indicated that rH5N6, ΔrH5N6, and rH5N8 were more pathogenic than rH5N1, and rH5N2 was less efficiently transmitted to contact chickens. These data suggest that N2 is likely not matched to the clade 2.3.4.4. HA and thus may explain why 2.3.4.4 H5N2 subtype viruses are not prevalent in China.

The rH5N6 virus was highly pathogenic to contact chickens, which is consistent with previous studies (Jiao et al., 2016). In addition, a virus created with short stalk NA showed adaptation and virulence enhancement of waterfowl influenza viruses among chickens (Munier et al., 2010). The ΔrH5N6 virus also replicated effectively in MDCK and CEF cells. Thus, our results suggest a possibility that high mortality of H5N6 with long-stalk NA on hosts limits the transmission and epidemics in chickens. Chickens inoculated with rH5N8 had severe infections, yet this virus was only poorly transmitted to contact chickens. The H5N8 virus occurs sporadically in reassortment events, but does not circulate as widely as the H5N1 subtype virus in China. However, the clade 2.3.4.4 H5N8 virus has spread around the world along two main long-distance migration routes and genetic analyses have shown close phylogenetic relationships among the H5N8 viruses in Asia and North America. This suggests future outbreak possibilities for the 2.3.4.4 H5N8 (Lycett et al., 2016).

In summary, we show that distinct NAs reassortments with clade 2.3.4.4 H5N1 subtype viruses are important for virus replication, pathogenicity, and transmission. The N6 and N8 NAs possess a fitness balance between HA and NA activity, which contributes to the high pathogenicity of H5N8 and H5N6 in mice and chickens. H5N2 virus doses not efficiently infect chickens by direct contact. H5Nx viruses constitute a threat to poultry and public health. Although a reassortant vaccine matched with clade 2.3.4.4 has been developed and used in poultry, more comprehensive surveillance work also should conducted.

## Author Contributions

YY, ZaZ, WQ, and ML conceived and designed the experiments; YY, ZaZ, and HL performed epidemiological investigation; YY, ZaZ, HL, XW, BL, XR, ZhZ, XZ, SL, and PH performed the animal experiments; YY, ZaZ, WQ, and ML contributed analysis; YY, ZaZ, WQ, and ML drafted the manuscript. All authors reviewed and revised the first and final drafts of this manuscript.

## Conflict of Interest Statement

The authors declare that the research was conducted in the absence of any commercial or financial relationships that could be construed as a potential conflict of interest.

## Footnotes

1 http://www.who.int/influenza/human_animal_interface/H5N1_cumulative_table_archives/en/

2 http://www.who.int/en/
